# Heart Rate Variability during Auricular Acupressure at Heart Point in Healthy Volunteers: A Pilot Study

**DOI:** 10.1155/2022/1019029

**Published:** 2022-04-25

**Authors:** Dieu-Thuong Thi Trinh, Que-Chi Thi Nguyen, Minh-Man Pham Bui, Van-Dan Nguyen, Khac-Minh Thai

**Affiliations:** ^1^Faculty of Traditional Medicine, University of Medicine and Pharmacy at Ho Chi Minh City, Ho Chi Minh City 100000, Vietnam; ^2^University Medical Center Ho Chi Minh City, University of Medicine and Pharmacy at Ho Chi Minh City, Ho Chi Minh City 100000, Vietnam; ^3^Thong Nhat Hospital, Ho Chi Minh City 100000, Vietnam; ^4^Faculty of Pharmacy, University of Medicine and Pharmacy at Ho Chi Minh City, Ho Chi Minh City 100000, Vietnam

## Abstract

Heart rate variability (HRV) is the variation in time between each heartbeat. Increasing HRV may contribute to improving autonomic nervous system dysfunctions. Acupuncture stimulation through the vagus plexus in the ear is considered as a method that can improve HRV. In this pilot study, we examined 114 healthy volunteers at the Faculty of Traditional Medicine, University of Medicine and Pharmacy at Ho Chi Minh City, from January to May 2020. During a 20-minute interval, participants were stimulated two times at the acupoint in the left ear with Semen seed. The heart rate and HRV values were monitored before, during, and after acupressure every 5 minutes. When we compared the experimental group with the control group, HRV significantly increased in the stage of ear-stimulated acupressure compared with the stage before and after the auricular acupressure (*p*=0.01, *p*=0.04, *p*=0.04 and *p*=0.02) and the difference was not statistically significant compared with the phase of nonstimulated (*p*=0.15, *p*=0.28). The changes in other values including SDNN (standard deviation of the average NN), RMSSD (root mean square of successive RR interval differences), LF (low-frequency power), and HF (high-frequency power) in all stages were not statistically significant (*p*=>0.05) between groups. Based on the results, we can determine the increase in HRV when conducting auricular acupressure with stimulation at the heart acupoint on the left ear. This leads to a direction in further studies for clinical application for patients with autonomic nervous disorder.

## 1. Introduction

The time interval between two consecutive heartbeats is called the heart interval, and the difference in each interval produces heart rate variability (HRV) [1, 2]. HRV is measured in milliseconds (ms). Heart rate variability (HRV) is an index influenced by various factors such as age, sex, physique, health status, the frequent use of alcohol, tobacco, certain drugs, and physiological conditions of circadian rhythms and contextual factors when measured. Therefore, HRV is considered as a proxy for the health status of the whole system [3]. Many studies have shown that chronic low HRV values are associated with sudden cardiac deaths, depression, and diabetic neuropathy. Therefore, improving HRV may contribute to the improvement of the related diseases [4–6].

The HRV measurement standards were developed by the European Society of Cardiology (ESC) and the North American Society of Cardiac Rhythm and Electrophysiology (NASPE) in 1996 and have become a popular measurement standard to this day. Among these standards, two methods of measuring time domain and frequency domain with HRV components are commonly applied in many studies on HRV [7–11]. Through HRV components, it is possible to assess sympathetic and parasympathetic activities of the heart.

Besides ECG, which is used as the gold standard to measure HRV, nowadays with the development of science and technology, many new methods have been developed to measure HRV such as photoplethysmography (PPG) through smartphone, smartwatch, ear strap, chest strap, or wrist strap devices. These methods allow for more convenient and cost-effective HRV monitoring [12]. Studies have suggested that the PPG method is equivalent to ECG, with a high correlation coefficient [13, 14]. Kyto HRM 2511B, a compact, wearable device in the ear, allows for simpler HRV measurements in comparison to ECG [13]. While ECG is considered to be the gold standard, using devices with PPG technology has become more popular.

In traditional medicine, acupuncture in the vagus nerve (i.e., a part of the autonomic nervous system) distribution areas in the ear is a method that affects HRV by increasing the parasympathetic activity and contributes to an increase in HRV in a beneficial way [9]. Accordingly, the middle of the ear cavity is considered to be the place where most of the vagus nerve is located, which is comparable to the heart acupoint [4]. This area is expected to be highly effective and of low risk, especially for the left ear canal. However, research on the effect of HRV when stimulating the heart acupoint in the ear alone is still limited [2]. Moreover, these studies are about needling acupoints in the ear and there has been no study that uses an ear seed—a tiny device that stimulates acupoints by pressing without using needles and that patients can use by themselves.

Therefore, this study was set out to evaluate how the use of ear seed at the heart point affects the autonomic nervous system through HRV value. Along with time-domain and frequency-domain measurements, we are also seeking for whether there are any changes during the auricular acupressure process? Furthermore, this study also investigates the undesirable events when doing auricular acupressure at the heart acupoint. The study is expected to form the basis for using auricular acupressure to improve HRV to treat related diseases in further studies.

## 2. Materials and Methods

Participants were healthy volunteers who lived in Ho Chi Minh City. The research ethics was approved by the Medical Ethics Council of the University of Medicine and Pharmacy at Ho Chi Minh City.

Volunteers would sign an informed consent form before the study. Participants were randomly assigned into two groups by the GraphPad software version 9.1. Participants in the experimental group received auricular acupressure in the left heart acupoint, while the control group received placebo auricular acupressure by removing the ear seed but keeping the sticker attached in the left heart acupoint. The study was designed as a single-blinded pilot study. Only participants were blinded and did not know which groups they would belong to.

The sample size *n* is calculated according to the formula:

(1) $$\begin{array}{c} n=\;\frac{{\left(z_{1-\beta }+z_{1-\alpha /2}\right)}^{2}.\sigma^{2}}{d^{2}}, \end{array}$$

where *n* is the number of sample sizes needed for the study; *z*_1 − *I*_ = 0.83; and *z*_1 − *α*/2_ = 1.96. According to Clancy J. A [7], *d* = 70.35 and *σ* = 178.65. With a 10% expected loss, *n* was calculated as 57. So the total sample of the study was 114.

### 2.1. Inclusion Criteria

The inclusion criteria included healthy men and women with no history of cardiovascular diseases, diabetes, and thyroid aged between 20 and 29 and had vital signs within the normal range (pulse, regular heart rate, and resting heart rate: 60–100 beats/min; resting blood pressure: from 90/60 mmHg to ≤140/90 mmHg; breathing rate: 16 ± 3 times/minute; temperature: 36.6–37.5°C; and SpO_2_ ≥ 95%). All volunteers had body mass index (BMI) from 18.5 to 23 kg/m_2_ and had no psychiatric stress problem during acupuncture day (confirmed by answering the DASS21 questionnaire with stress point less than 15 points).

### 2.2. Exclusion Criteria

The exclusion criteria included volunteers whose ages were out of the range above used stimulants such as beer, alcohol, coffee, and tobacco within 24 hours before conducting the study. No volunteers played sports 2 hours before the study or had skin injuries in the area of auricular acupressure. Women who were in menstruation period, pregnancy, or breastfeeding, people using drugs affecting blood pressure, and heart rate within one month were also excluded.

### 2.3. Criteria to Stop Research

The criteria were participants who wanted to stop participating in the study or had overreacted parasympathetic stimulation symptoms such as dizziness, nausea, vomiting, pain, and allergy at the stimulus area. These cases would be recorded as unexpected events.

### 2.4. HRV Measurement

Monitoring values in the periods included before, during, and after auricular acupressure by using Kyto HRM-2511B, a photoplethysmography device, which was attached to the right earlobe of the participants. Monitored values included heart rate, heart rate variability (HRV consists of changes in the time intervals between consecutive heartbeats or between two successive R-waves of the QRS signal on the electrocardiogram-RR intervals, and HRV is measured in milliseconds), time-domain components SDNN (standard deviation of RR intervals), RMSSD (root square root of mean squares of differences between RR intervals), frequency-domain LF (low-frequency range—LF has a frequency of 0.04–0.15 Hz), and HF (high-frequency band—HF has a frequency of 0.15–0.4 Hz).

Auricular acupressure: we conducted auricular acupressure at the heart acupoint on the left ear, which was located in the middle of the ear cavity by using a sticker with Vaccaria ear seed (experimental group) or a sticker without seed (control group) for 20 minutes with two times of stimulating. The time of stimulation was 30 seconds with two acupressure movements per second, resulting in a total of 60 acupressure movements per stimulation.

**Figure 1:**
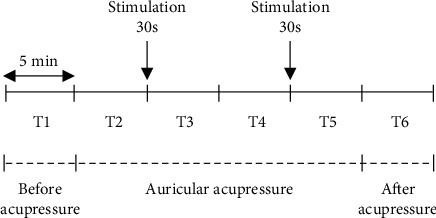
Study protocol. T1: before auricular acupressure, T2: auricular acupressure without stimulation, T3: the 1st auricular acupressure with stimulation in 30 sec, T4: auricular acupressure without stimulation, T5: the 2nd auricular acupressure with stimulation in 30 sec, and T6: after auricular acupressure.

HRV was monitored every 5 minutes. The measurement profile and measurement times (T1-T6) are schematically shown in Figure 1.

### 2.5. General Protocol

The study was conducted in a quiet room from 8 : 00 to 10 : 00 A.M. at 26 ± 1°C. Participants rest for 10 minutes, and then, their pulse rate, heart rate, blood pressure, breathing rate, and SpO_2_ were measured. Participants did not speak and did not change posture during acupressure.

### 2.6. Statistical Analysis

Data were analyzed using SPSS version 22.0. *T*-test was used to compare baseline characteristics and heart rate of the volunteers between groups for each stage. HRV and HRV components (SDNN, RMSSD, LF, and HF) at the time before acupressure, during acupressure, and after acupressure in each group were compared by the Wilcoxon signed-rank test and between two research groups by the Mann-Whitney *U* test. The results were statistically significant when *p* < 0.05.

## 3. Results

### 3.1. General Characteristics of the Study Population

Table 1 shows the general characteristics of the study population in each group at the beginning of the experiment. The anthropometric and hemodynamic data were in their normal range and did not show significant differences between groups. There were no significant differences in sex and age between the experimental and control group (*p* > 0.05). The difference in basic characteristics of the experimental and control groups was not significant (*t*-test, *p* > 0.05, Table 1). Pulse, heart rate, blood pressure, respiratory rate, SpO_2_, and BMI of all participants were within normal values, which is required for the safety of participants.

When comparing values of an index between groups including pulse, heart rate, blood pressure, respiratory rate, SpO_2_, and BMI, the results were not statistically significant with *p* > 0.05. This shows the random distribution of participants into two groups, thereby ensuring accuracy and objectivity when comparing the two groups.

### 3.2. Heart Rate and HRV in Each Stage of the Study

The heart rates in each stage of the two study groups are shown in Table 2. There was no statistically significant difference in heart rate in each stage of T1, T2, T3, T4, T5, and T6 between groups (*p* > 0.05, *t*-test).

**Figure 2:**
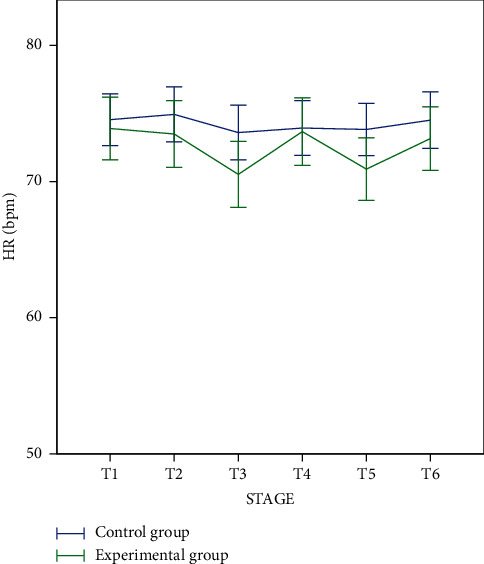
Heart rate and HRV in each stage. (a) Heart rate. (b) HRV.

In the experimental group, heart rate in the stage of auricular acupressure with stimulation was lower than that of before, after, and in the stage without stimulation (Figure 2(a)). HRV in the stage of auricular acupressure with stimulation was greater than HRV before and after acupressure but was not statistically significantly different from HRV in the stage of auricular acupressure without stimulation (Figure 2(b)).

In the control group, the HRV difference between stages was not statistically significant (Figure 2(b)).

### 3.3. Auricular Acupressure at Heart Acupoint Alters Elements of HRV

The variation of the time domain and frequency domain is shown in Table 4. There were no significant differences in the SDNN and RMSSD between groups (Wilcoxon signed rank-sum test, *p* > 0.05). There were no significant differences in the LF and HF between groups in each stage (Wilcoxon signed rank-sum test, *p* > 0.05).

## 4. Discussion

With the expectation of investigating how the use of ear seed at the heart point affects the autonomic nervous system through HR, HRV, SDNN, RMSSD, LF, and HF values of 114 volunteers, we suggest that auricular acupressure on the point that is in the distribution of vagus nerve can have a significant affect to the autonomic cardiovascular system on healthy people.

### 4.1. Heart Rate

The first value to be investigated when stimulating the heart auricular acupoint in the ears was the heart rate. In this study, the heart rate in the experimental group was not statistically significant compared with the control group (Table 2). This result is similar to the study of Gao et al. on healthy volunteers. When performing stimulated acupressure of the vagus nerve in the ear, the heart rate was reduced [8, 15–17]. The decrease in heart rate is a precedent for further research on heart rate variability.

### 4.2. HRV

As for the experimental group, the HRV value increased in the stage of auricular acupressure with stimulation compared to the other stages (Table 3). This result is similar to the study of Clancy et al. [7, 8]. However, when compared to the control group, the increased HRV in the experimental group is only statistically significant in the stimulation stage compared with that of the before and after acupressure. The difference was not statistically significant compared to the nonstimulated acupressure stage (*p* > 0.05) (Figure 2(b)). Meanwhile, the heart rate decreased in both the nonstimulated and stimulated acupressure stage compared with that before and after the acupressure. According to the HRV definition, when the heart rate drops, it creates more space for variation between consecutive heart rates, leading to higher HRV. However, in our study, when the heart rate decreased, there was no difference in the stimulated acupressure stage compared with the nonstimulated acupressure stage in HRV. The question is as follows: is there any conflict between heart rate and HRV values in our study?

It is known that HRV is not only physiologically linked to heart rate via autonomic nerve activity but also mathematically linked [18]. In experimental studies, it has been shown that at least one part of the HRV value is influenced by the intrinsic factor of the sinus node, namely, the cyclic length of myocardial cells or the period of RR that has a nonlinear relationship with neurotransmitter concentrations at the sinus node [19, 20]. This leads to the situation that with the same intensity of parasympathetic activity, a longer interval between RRs leads to a higher HRV [20, 21]. Therefore, although parasympathetic activity lowers the heart rate, HRV changes may be statistically insignificant because HRV also depends on neurotransmitters affecting the length of RR interval between heart cycles.

The relationship between the RR distance and heart rate mentioned in the SACHA study shows that when the heart rate is of the same value, the difference between the RR intervals may not be equal, leading to different HRV [22]. This assumption may partly explain the mismatch between the reduction in heart rate and the increase in HRV observed in our study.

The increase in HRV when stimulating heart auricular acupoint can be a potential approach in clinical practice. Previous studies have shown that stimulating the vagus nerve in the ear with an increase in HRV will have an antiarrhythmic effect, potentially reducing the recurrence of atrial fibrillation when combined with an antiarrhythmic drug [23, 24]. Therefore, future studies to determine the influence of auricular acupuncture on heart acupoints in patients with atrial fibrillation are particularly promising.

### 4.3. The Variation of Time Domain

#### 4.3.1. SDNN

The SDNN is the standard deviation of normal sinus rhythms, measured in milliseconds (ms). In 5 minutes, the SDNN values were mainly influenced by parasympathetic-mediated respiratory sinus arrhythmia (RSA) [3].

In our study, the SDNN change was not statistically significant (*p* > 0.05) at the study stages and in both the experimental group and the control group. Nevertheless, in the study of Boehmer et al. [15], SDNN increased after performing auricular acupuncture. To clarify this difference, it is important to compare the inclusion criteria and methods of the two studies. In the study of Boehmer, the participants were young men (23 ± 2 years old), who were regularly engaged in moderate-intensity physical activity over the past 12 months (7 ± 3 hours/week). When conducting the study, participants were performed auricular acupressure at the heart acupoint and measured HRV in the supine position and then switched to a standing position to measure HRV. Thereby, it can be proposed that, in Andreas' study, a baroreceptor reflex occurred in participants with high sensitivity, leading to an increase in RSA [25]. An increase in RSA leads to an increase in SDNN.

The SDNN is considered the “gold standard” in cardiovascular risk stratification, especially in the 24-hour recordings [26]. The SDNN value predicts morbidity and mortality. The study by Kleiger RE et al. based on 24 hours of ECG monitoring and HRV analysis showed that cardiovascular patients with SDNN values less than 50 ms are classified as unhealthy, 50–100 ms corresponds to harmed health, and over 100 ms is classified as healthy. Poststroke patients with SDNN values above 100 ms had a 5.3-fold lower risk of death compared with patients with values below 50 ms [27]. Whether increasing SDNN can reduce mortality risk is being studied.

#### 4.3.2. RMSSD

When surveying RMSSD through each stage, we noted that RMSSD was different with no statistical significance (*p* > 0.05) between both groups, which was also observed by Andreas et al. [15].

Considering the time-domain HRV, it was found that RMSSD is affected more by parasympathetic activity rather than SDNN. While compared with the frequency-domain index, RMSSD was correlated with HF; however, RMSSD was less affected by respiratory frequency than HF. It can be, therefore, suggested that RMSSD is the main time-domain index used to estimate changes in parasympathetic activity through HRV [28]. However, in our study, parasympathetic activity will increase, but the variation in RMSSD is not statistically significant. The reason is that although RMSSD reflects parasympathetic activity in the heart, it is an indirect result and a major factor in the alteration of *R* peaks produced by the sinus node. The activity is also influenced by receptors on the sinus node cells [29].

### 4.4. The Frequency-Domain Spectral Analysis

#### 4.4.1. Low-Frequency (LF) Power

In our study, if we compare the LF value at each stage, including before, during, and after acupressure, the difference is not statistically significant (*p* > 0.05) in both the experimental and control groups (Table 4). This result is similar to the studies of Shen et al. [24] and Lee et al. [10], but different from that of Gao et al. [7, 8, 15]. In the study of Gao, LF value was increased in both stages of stimulating the heart point with electroacupuncture (electric vibrating pen) and auricular needling (stimulating the acupuncture point with a needle).

Physiologically, the stimulation of the vagus nerve will increase the activation of baroreceptors and then activate the baroreflex. Many studies have shown that the LF value reflects the activity of the sympathetic nervous system and the efferent parasympathetic nervous system (A and C fibers), respectively, related to the action of baroreceptors. [30–33]. This explains the results of Gao where stimulation of the vagus nerve in the ear would increase the activation of baroreceptors, leading to an increase in LF.

Moreover, Stauss suggested that when stimulating the vagus nerve in the ear, the hemodynamic changes will depend on the number of stimulation sites, stimulation parameters (potential, frequency, length, pulse, and current direction), which lead to different changes in heart rate, blood pressure, and baroreflex [34]. In our study, we used Semen seeds to perform auricular acupressure, so the stimulation was weaker than that in Gao's study, which used electroacupuncture. Therefore, lower sensitivity and weaker baroreflex will lead to a change in LF value that is not statistically significant.

The baroreflex plays an important role in hemodynamic stability and cardiovascular protection and is also a strong prognostic factor in some cardiovascular diseases such as hypertension and chronic heart failure [35]. LF is considered an indicator of the sensitivity of the baroreceptor reflex; therefore, measuring the LF value is a noninvasive method to determine the sensitivity of the baroreceptor [36]. When stimulating the heart point with Semen seed, the LF value did not change.

#### 4.4.2. High-Frequency (HF) Power

Theoretically, stimulating the heart point in the ear belonging to the vagus nerve will increase parasympathetic activity. Accordingly, HF modulated by parasympathetic activity will increase. Nevertheless, in our study the HF value changed but the difference was not statistically significant between the stages, including before, during, and after atrial pressure in both groups (*p* > 0.05) (Table 4). Hayano and Yuda suggested that the HF value of HRV does not necessarily reflect cardiac parasympathetic functioning. In fact, when observing the heart rate oscillation through the autonomic nerve, the HF band is regulated by cardiac parasympathetic activity. HF is influenced by parasympathetic activity in the heart in the frequency range of 0.15–0.4 Hz. In addition, RSA is considered to be a determinant of the HF component. Even if HRV in the HF band decreases or disappears, it does not mean that cardiac parasympathetic nerve arrest or autonomic dysfunction is occurring. This occurs when the respiratory rate is outside the HF range, such as in slow breathing HF < 0.15 Hz (9 breaths/min), during deep, or fast breathing HF > 0.4 Hz (24 breaths/min) [37]. Therefore, when conducting an investigation of the HF value to assess cardiac parasympathetic function, it is necessary to monitor the respiratory parameters during the survey [38, 39].

The HF components compared with other HRV frequency components such as LF and VLF are weak clinical prognostic factors in short-term HRV measurements [40]. It has been found that HRV observed in the HF band has data that are not necessarily mediated by autonomic nerves [37]. This phenomenon is described by various terms such as complex HRV, erratic sinus rhythm, or heart rate fragmentation (HRF) [41]. This is a type of instability characterized by an anomalous appearance between peaks in the RR time series even though the ECG shows sinus rhythm. The occurrence of HRF may confound the association between HF and cardiac parasympathetic function and distort the prognosis, so HF is rarely used in clinical evaluation [37]. However, low HF is often correlated with stress, anxiety disorders, and stress; therefore, improving the HF value has positive implications for body health [38].

#### 4.4.3. Unwanted Reactions

One case of drowsiness was recorded in the experimental group and not in the control group. This can be both an unwanted reaction and a beneficial effect. If this method is applied to patients with insomnia at the right time, there will be therapeutic benefits [15, 25, 26].

### 4.5. Limitations

First, the respiratory frequency had not been investigated during the study process, so the effect of parasympathomimetics on HF had not been accurately assessed.

Second, this is the first study to conduct acupressure to investigate HRV; hence, we performed it on healthy volunteers to ensure safety and to monitor possible dangerous cardiovascular events during acupressure. Evaluating results on healthy people will not represent the goal of clinical application with subjects who are cardiovascular patients. Therefore, in further research it will be focused on patients with chronic cardiovascular or HRV-related diseases.

## 5. Conclusions

The results of the study show that HRV value increased with stimulated acupressure at the heart acupoint of the left ear in healthy volunteers and had a high safety effect. This study is the first step to evaluate the safety of acupuncture, opening a direction in the future for traditional medicine studies on auricular therapy for patients with autonomic nervous disorders.

## Figures and Tables

**Table 1:** Anthropometric subjects' characteristics.

Characteristics	Experimental group (*n* = 57)	Control group (*n* = 57)	*p* value
	(Mean ± SD)		
Gender (*n*, %)			
Male	27 (47.37)	28 (49.12)	0.85^a^
Female	30 (52.63)	29 (50.88)	
Age (years)	25.54 ± 2.80	25.12 ± 2.63	0.41^b^
Pulse (bpm)	73.89 ± 8.67	71.88 ± 6.67	0.14^c^
HR (bpm)	73.89 ± 8.67	71.88 ± 6.67	0.14^c^
SBP (mmHg)	109.18 ± 10.99	107.39 ± 8.45	0.33^c^
DBP (mmHg)	72.91 ± 5.56	72.11 ± 4.80	0.41^c^
Breath (bpm)	16.26 ± 1.99	16.21 ± 1.86	0.88^c^
SpO_2_ (%)	97.09 ± 1.43	96.93 ± 1.45	0.56^c^
BMI (kg/m^2^)	20.28 ± 1.54	20.67 ± 1.51	0.17^c^

Note: a-Fisher's exact test, b-Mann-Whitney *U* test, *t*-test. HR = heart rate, SBP = systolic blood pressure, DBP = diastolic blood pressure, BMI = body mass index. SD: standard deviation.

**Table 2:** Heart rate behavior between groups.

Stage	Heart rate (bpm) (mean ± SD)		*p* value
	Experimental group (*n* = 57)	Control group (*n* = 57)	
T1	73.89 ± 8.67	71.88 ± 6.67	0.17
T2	73.49 ± 9.21	72.14 ± 6.98	0.38
T3	70.53 ± 9.11	71.07 ± 7.16	0.72
T4	73.67 ± 9.37	71.49 ± 7.13	0.17
T5	70.91 ± 8.66	71.72 ± 6.69	0.58
T6	73.16 ± 8.79	71.87 ± 7.29	0.40

SD: standard deviation. Table 3 shows the HRV of each stage between groups. There was a statistically significant difference in HRV between groups at the T1, T2, T4, and T6 stages (*p* < 0.05 Mann-Whitney *U* test). There was no statistically significant difference in HRV between groups in stages T3 and T5 (*p* > 0.05 Mann-Whitney *U* test).

**Table 3:** Heart rate variability in each stage between groups.

Stage	HRV (ms) (median (IQR 25th – 75th))				*p* value
	Experimental group (*n* = 57)		Control group (*n* = 57)		
T1	53.00	49.00, 57.50	57.00	51.00, 61.50	0.01
T2	53.00	47.00, 55.00	55.00	50.00, 63.00	0.04
T3	54.00	50.00, 58.00	57.00	50.00, 63.00	0.15
T4	52.00	47.50, 55.00	55.00	48.00, 61.50	0.04
T5	54.00	50.50, 59.50	56.00	51.00, 63.00	0.28
T6	52.00	49.00, 57.50	56.00	50.00, 63.00	0.02

IQR: interquartile range.

**Table 4:** The variation of time domain.

Stage	SDNN (median (IQR))			RMSSD (median (IQR))			LF (median (IQR))			HF (median (IQR))		
	Experimental group *n* = 57	Control group *n* = 57	*p* value	Experimental group *n* = 57	Control group *n* = 57	*p* value	Experimental group *n* = 57	Control group *n* = 57	*p* value	Experimental group *n* = 57	Control group *n* = 57	*p* value
T1	42.97 (33.19, 53.52)	42.71 (34.10, 55.84)	0.90	30.85 (19.51, 51.33)	32.41 (22.43, 46.10)	0.90	321.23 (167.65, 544.03)	290.37 (168.71, 680.75)	0.90	292.81 (131.04, 568.82)	281.96 (147.09, 519.95)	0.90

T2	43.65 (27.68, 53.84)	51.02 (34.89, 58.44)	0.65	29.11 (18.35, 49.64)	28.82 (21.70, 50.53)	0.65	292.14 (184.77, 562.70)	336.65 (169.43, 603.96)	0.65	278.62 (141.36, 545.21)	272.03 (161.88, 434.44)	0.65

T3	42.97 (26.65, 54.31)	44.81 (33.14, 54.79)	0.98	32.56 (19.42, 49.80)	34.47 (24.85, 53.97)	0.98	301.54 (176.90, 585.02)	318.40 (156.40, 634.21)	0.98	223.11 (113.14, 516.47)	270.55 (176.56, 496.28)	0.98

T4	45.30 (37.71, 57.54)	46.30 (40.15, 54.43)	0.82	31.24 (19.93, 51.26)	33.32 (22.10, 50.75)	0.82	308.70 (173.52, 593.46)	322.61 (174.94, 659.84)	0.82	269.36 (139.44, 554.53)	279.77 (186.05, 489.68)	0.82

T5	42.04 (29.60, 51.60)	46.16 (35.37, 54.62)	0.35	31.94 (17.24, 54.68)	31.28 (25.75, 50.46)	0.35	283.66 (146.31, 511.77)	364.90 (173.39, 609.40)	0.35	287.66 (153.18, 501.74)	316.51 (215.40, 555.57)	0.35

T6	45.75 (30.66, 55.94)	47.07 (37.20, 61.61)	0.29	27.52 (20.86, 47.27)	28.39 (21.66, 49.96)	0.29	255.83 (162.03, 513.95)	325.11 (161.59, 681.28)	0.29	252.31 (115.47, 597.09)	343.77 (219.05, 631.33)	0.29

IQR: interquartile range.

## Data Availability

The data used in this research can be obtained from the corresponding author.
